# Antioxidant Activity of *Humulus lupulus* Phenolic Hop Extracts in Creating a New Pâté: An Element Affecting Fat Stability and Microbiological Quality during Storage

**DOI:** 10.3390/molecules29071561

**Published:** 2024-03-31

**Authors:** Agnieszka Bilska, Joanna Kobus-Cisowska, Janusz Wojtczak, Ryszard Kowalski, Ewelina Kaczmarek

**Affiliations:** 1 Department of Meat Technology, Poznań University of Life Sciences, Wojska Polskiego 28, 60-637 Poznan, Poland; agnieszka.bilska@up.poznan.pl (A.B.); ryszard.kowalski@up.poznan.pl (R.K.);; 2 Department of Gastronomy Sciences and Functional Food, Poznan University of Life Sciences, Wojska Polskiego 28, 60-637 Poznan, Poland; 3 Department of Animal Breeding and Product Quality Assessment, Faculty of Veterinary Medicine and Animal Science, Poznan University of Life Science, Wojska Polskiego 28, 60-637 Poznan, Poland; janusz.wojtczak@up.poznan.pl

**Keywords:** hop extracts, liver pâté, peroxide number, TBARS, redox potential, microbiological quality

## Abstract

The aim of this study was to evaluate the effect of hop extracts on changes in the primary and secondary fat oxidation products, physicochemical properties, and microbiological quality of pâté-type offal sausages obtained through the partial replacement of animal fat with vegetable fat. This study demonstrated that the extraction efficiency varied among hop cone varieties, with the highest efficiency observed for the Lubelski variety and the lowest for the Magnum variety. The phenolic compound content was higher in the Magnum cones (2.74 ± 0.11 mg/g dry matter) compared to the Lubelska cones (2.27 ± 0.05 mg/g of product). Additionally, the DPPH radical scavenging activity was greater in the extract from the Magnum cones (4.21 ± 0.09 mg TE/g d.w.) than in the extract from the Lublelski cones (3.87 ± 0.05 mg TE/ g d.w.). Similarly, the extracts from the Lubelski cones exhibited a higher antiradical activity against the ABTS radical compared to the extract from Magnum cones. Throughout storage, a significant increase in the pH value was observed in the control sample and in the samples with a 20% replacement of animal fat with rapeseed oil and Magnum hop extract. However, the addition of Lubelski hop extract resulted in a decrease in the pH value during the 15-day storage period. The samples with a 20% replacement of animal fat with rapeseed oil and 0.1% Lubelski hop extract showed the least changes in water activity during storage. The samples with a 20% replacement of animal fat with rapeseed oil and the addition of 0.2% Lubelski hop extract had the lowest peroxide value and TBARS index throughout the storage period. The addition of hop extract inhibited the growth of the total number of microorganisms in the tested sausages. In the samples with a 20% replacement of animal fat with rapeseed oil, the content of aerobic microorganisms, compared to the control sample, was statistically significantly lower.

## 1. Introduction

Consumers are increasingly paying attention to the quality of food products and are looking for products that are more desirable from a dietetic and rational nutrition point of view. Therefore, manufacturers of offal meats, in order to meet consumer demands, must make changes to the formulation of traditional meats by improving the PUFA/SFA (polyunsaturated fat acid/saturated fatty acids) ratio and n-6:n-3 PUFA and by adding vegetable ingredients, which are a rich source of active biological substances.

A good way to improve the quality and fatty acid composition profile of meat products without radically changing eating habits can be to partially replace animal fat with vegetable fat. However, it should be remembered that fat from slaughtered animals has a beneficial effect on the texture of the finished product and its juiciness and palatability. Therefore, replacing it with vegetable fat is not so easy without changing selected quality characteristics of the finished product [1]. The introduction of unsaturated fatty acids, mainly polyunsaturated, can lead, among other things, to a deterioration in the oxidative stability during storage of cured meats. Fat oxidation can be reduced by, among other things, the addition of antioxidants, which include all substances which inhibit reactions with oxygen [2]. One of the most important functions of antioxidants is to trap free radicals, large amounts of which, in the body, can cause diseases such as cardiovascular diseases, diabetes, cancer, and chronic respiratory diseases [3,4,5]. To date, rosemary extract, which has antimicrobial as well as antioxidant effects, has been used extensively, but natural substances are being sought that can prevent fat oxidation in animal products [6,7].

Hop cones contain a wealth of secondary plant metabolites. The primary compounds present in the fully developed female inflorescences of *Humulus lupulus* include hop bitter acids, terpenes, and chalcones. Additionally, they are rich in flavonol glycosides such as rutin, kemferol, quercetin, and quercitrin as well as catechins like catechin gallate and epicatechin gallate [4,8,9]. The essential oil of hops contains many volatile constituents, including monoterpenes (myrcene) and sesquiterpenes (β-caryophyllene, farnesene, humulone), which, together, account for 57–82% of the total content. The quantity of these chemicals varies depending on the cultivar and the methods of detection used.

The cones of female hop plants are now used almost exclusively as an ingredient for the brewing industry, since dried cones and hop preparations (e.g., pellets, extracts) are essential for the characteristic bitterness and aroma of beer [10,11].

A natural antioxidant is hop extract, which, in addition to its antioxidant properties, possesses calming and strengthening properties and is, therefore, used in pharmacological preparations. Hops owe their antioxidant properties to polyphenols. These compounds can reduce peroxides and block free radicals. In addition, they can also form complexes with metals, which are responsible for catalyzing oxidation reactions, and inhibit the activity of enzymes that cause fat oxidation (e.g., lipoxygenases) [4]. This compound stands out for its broad spectrum of biological activities. It not only has antioxidant properties but also antibacterial, antiviral, and antifungal properties [4,12]. Due to the wide range of biological activities of the compounds contained in hops, they are also being investigated as potential antioxidants. Yamaguchi et al. [13] demonstrated in vitro antioxidant properties for hop bitter acids and xanthohumol. Their study used the ORAC (Oxygen Radical Absorbance Capacity) method commonly used to assess antioxidant capacity in biological samples and foods. The highest ORAC value corresponds to the highest antioxidant activity. Polyphenon 60, which contains catechins sourced from green tea, served as the control with the highest ORAC value among edible plants. It was noted that the overall ORAC value for xanthohumol was similar to that of Polyphenon 60 and notably higher compared to the values of vitamins E and C [4,13].

The objective of this research was to assess how the incorporation of hop extracts influences alterations in the physicochemical attributes, stability of the fat component, and microbiological safety of pâtés formulated with 20% rapeseed oil.

## 2. Results and Discussion

### 2.1. Characteristics of Hop Extracts

The extraction efficiency varied depending on the raw materials tested (Table 1). It was demonstrated that a higher extraction yield was observed for the extract of Lubelski variety cones, whereas the lowest yield was obtained for the extract of Magnum variety hop cones. The extracts tested were also evaluated for their content of total polyphenols. These compounds are antioxidants whose composition and proportions of occurrence determine the total antioxidant activity of the selected raw material. The tested extracts contained a significant amount of polyphenols in their composition. A higher content of phenolic compounds was found in the extracts from cones of the Magnum variety (2.74 ± 0.11 mg/g d.w.), and a lower content was observed for the Lubelski variety (2.27 ± 0.05 mg/g d.w.). In the hop extracts, chlorogenic acid was the most dominant phenolic compound, which ranged around 191.41 μg/g d.w. in the Lubelski samples and 768.32 μg/g d.w. in the Magnum extract. There were no differences in terms of the content of ferulic acid, vanillic acid, o-coumaric acid, and p-coumaric acid in the extracts tested. Among the flavonols, epicatechin, rutin, and quercetin were dominant.

The tested extracts were also evaluated for their antioxidant potential with DPPH and ABTS tests. It was found that the extracts made from the hop cones tested differed in their properties. The extract from the Magnum cones scavenged DPPH radicals at a level of 4.21 ± 0.09 mg TE/g d.w., while the DPPH radical scavenging activity for the extract from the Lubelski variety was lower, at 3.87 ± 0.05 mg TE/g d.w. This study also confirmed a high activity with the ABTS radical: a higher activity was found for the extracts from the Lubelski cones, and a lower activity was found for the Magnum ones. The ABTS (2,2’-azino-bis(3-ethylbenzothiazoline-6-sulfonic acid)) and DPPH (2,2-diphenyl-1-picrylhydrazyl) assays are both methods commonly used to measure the antioxidant activity of compounds [14,15,16]. While they both assess the ability of antioxidants to scavenge free radicals, they differ in their mechanisms and reaction kinetics, which can lead to variations in their results. These differences may be due to differences in the mechanisms of free radical generation, their reactivity with polyphenols, and test conditions. Additionally, different polyphenols may have different chemical structures and properties, which may affect their ability to interact with different types of free radicals. For example, polyphenols containing hydroxyl groups may have strong antioxidant properties, but their ability to neutralize different types of free radicals may vary [17,18,19]. 

The action of polyphenols in the food matrix can be activated and used according to many options [20,21,22,23,24]. The literature on this subject indicates that polyphenols can directly inhibit and scavenge free radicals from the matrix or may act indirectly by affecting the enzymes responsible for the formation of radicals [21,22,23,24]. Research has repeatedly confirmed that the use of polyphenols in food can inhibit oxidative changes in animal and vegetable fats [25]. However, there is no information on the effect of hop extract on the stabilization of the fat fraction in meat products.

### 2.2. Evaluation of the Effect of the Addition of Hop Extracts on Changes in the Physicochemical Characteristics of Experimental Sausages

In this study, the basic chemical composition of offal sausages of the pâté type was determined, i.e., protein, fat, water, and salt contents. The fat content was in accordance with PN-A-82007:1996/Az1:1998 [26] and did not exceed 60%. The average fat content of the tested sausages was 40.23% in the control sample and 42.59% in the samples with a 20% substitution of fine animal fat with rapeseed oil. A similar relationship was observed in the study by Martín-Sánchez et al. [27], in which the addition of rapeseed oil increased the fat content of the finished product. In contrast, the salt content was determined to be 1.52% in all the samples in our study.

The measurement of hydrogen ion concentration (pH) is the most common test used to assess the quality of meat and meat products. During 15 days of storage, the control sample had the highest pH value. A statistically significant increase in the pH value during storage was observed in the control sample and in the samples with a 20% replacement of animal fat with rapeseed oil and the addition of Magnum hop extract (Table 2). In contrast, the addition of Lubelski hop extract resulted in a decrease in the pH values during the 15 days of storage in the experimental sausages. The desired pH range can vary depending on the type of meat product and the specific processing techniques employed. For example, in certain types of processed meats, like fermented sausages or dry-cured meats, a slightly higher initial pH may be desirable to facilitate the growth of beneficial microorganisms or achieve specific flavor profiles [28,29]. Conversely, an excessively high pH level in meat can indicate a poor quality or improper handling. High pH values may result from factors such as prolonged post mortem storage, inadequate chilling, or stress conditions in the animal prior to slaughter. Elevated pH levels can lead to undesirable meat characteristics, such as a darker color, a softer texture, a reduced water-holding capacity, and increased susceptibility to spoilage. Therefore, while a decrease in pH is generally desirable for fresh meat products, the optimal pH range can vary depending on the specific requirements of different meat products and processing techniques. Monitoring and controlling pH levels are crucial steps in ensuring the quality, safety, and shelf-life of meat products throughout the production process [29].

From this study, it was found that the redox potential increased in all the samples during cold storage (Table 2). It was noted that the values of the oxidation reduction potential of the samples with a 20% replacement of animal fat with rapeseed oil and with the addition of hop extracts, throughout the storage period, were at a significantly (*p* < 0.05) lower level compared to the control sample. 

Water activity affects a number of characteristics, including appearance, aroma, texture, palatability, and also a product’s susceptibility to spoilage. Therefore, controlling the optimum water activity enables one to achieve the highest quality and maximum shelf-life of a product [30]. In the conducted study, the water activity levels varied between 0.936 (on the fifteenth day post production, for the sample incorporating the substitution of animal fat with rapeseed oil and the addition of Magnum hop extract at 0.1%) and 0.964 (immediately after production, for the control sample). The sample displaying the least fluctuations in water activity during storage was the one supplemented with 0.1% Lubelski hop extract, in conjunction with a 20% replacement of animal fat with rapeseed oil.

### 2.3. Evaluation of the Effect of the Addition of Hop Extracts on the Stability of the Fat Fraction in Experimental Sausages

Oxidative changes in lipids were analyzed in the experimental sausages during cold storage by monitoring changes in the content of primary (peroxide number—PV) and secondary oxidation products (TBARS). The peroxide number value increased statistically significantly in the experimental samples during the 15 days of storage (Table 3). 

However, the trials with a 20% replacement of animal fat with rapeseed oil and the addition of hop extracts showed a lower increase in peroxide number throughout the storage period, compared to the control trial. A similar relationship was observed by Bilska et al. [31,32] in pâté-type offal sausages with added rapeseed oil, showing that the control sample without added antioxidant substances had a higher peroxide content during storage compared to the samples with added oil and antioxidants. Among the tested sausages, the sample containing 0.2% Lubelski hops extract exhibited the least variance in the peroxide number values between 1 and 15 days post production, with a difference Δ = 0.15. The values of the directional coefficients in the experimental sausages were lower compared to the control sample. The lowest value was obtained for the sample with 0.2% Lubelski hop extract, which may indicate that the level of additive used showed the best antioxidant effect. 

The composition of fatty acids, the presence of prooxidants and antioxidants, and also the storage conditions affect the rate of oxidative changes in fat [33]. A method for monitoring secondary fat oxidation products in meat and meat products is TBARS. In our study, we found that, during the storage of cured meats, the growth dynamics of secondary lipid oxidation products in the samples with a 20% substitution of animal fat with rapeseed oil were lower compared to the control sample. Similar to the determination of the peroxide number, the sample with a 20% animal fat substitution with rapeseed oil and the addition of 0.2% Lubelski hop extract (Δ = 0.41) was characterized by the lowest TBARS value throughout the storage period. Changes in the malondialdehyde content of the experimental sausages during the 15 days of storage are summarized in Table 3. 

Polyphenols have a significant impact on the fat oxidation process in pâtés. Fat oxidation processes are the main cause of deterioration in the sensory quality and durability of pâtés. Research suggests that the presence of polyphenols can effectively delay this process through several mechanisms of action. Firstly, polyphenols neutralize the free radicals that are formed during fat oxidation, which inhibits further oxidation reactions. Moreover, polyphenols can bind metal ions, which are the catalysts for fat oxidation processes, and inhibit the activity of the peroxidase enzymes responsible for these processes. Additionally, research suggests that polyphenols may reduce the formation of lipid aldehydes, which are one of the main byproducts of fat oxidation. As a result, the presence of polyphenols in pâtés may be beneficial to maintaining their sensory quality and durability as well as to the health of consumers, by preventing the formation of harmful compounds during fat oxidation [6,34,35].

### 2.4. Evaluation of the Effect of the Addition of Hop Extracts on the Microbiological Safety of Experimental Sausages

During the refrigerated storage of meat and meat products, due to the development of aerobic microflora, the activity of tissue and bacterial enzymes, the oxidation of haem pigments, the oxidation of lipids, and also the drying of the surface due to water evaporation, their quality and shelf-life deteriorate [36]. The microbiological quality of the experimental sausages was assessed by determining the total microbial, enterococci, Enterobacteriaceae, and *Pseudomonas* counts. 

The tests carried out showed that the aerobic microbial content of the control sample and the 20% OR sample was at 5 log_10_ cfu/g on the last test date. In contrast, in the samples with 20% animal fat replacement with rapeseed oil and the addition of hop extracts, the total plate count did not exceed 3.43 log_10_ cfu/g during the 15 days of storage (Table 4). The smallest increase in the total plate count was observed in the sample with the addition of 0.2% Lubelski hop extract.

Pseudomonas bacteria are included in the saprophytic microflora and are known for causing spoilage in meat products when stored under aerobic and refrigerated conditions. They exhibit the capability to produce extracellular enzymes such as lipases and proteinases even at low temperatures [37,38]. The presence of these enzymes leads to irreversible alterations in the quality of meat and the development of an undesirable odor when the count of Pseudomonas bacteria exceeds 10^7^–10^9^ colony-forming units per gram (cfu/g) [37,39]. In the conducted study, the Pseudomonas counts were assessed to be between 10^2^ and 10^3^ colony-forming units per gram (cfu/g) (refer to Table 4). Furthermore, no Enterobacteriaceae or heat-resistant enterococci were detected in the experimental sausages throughout the 15-day storage period. Comparable findings were observed in the assessment of the microbiological quality of vacuum-packed pâtés stored for 28 days at +4 °C [40].

*Humulus lupulus* polyphenols are natural chemical compounds that have antimicrobial properties against various types of bacteria, viruses, and fungi. Their mechanism of action on microorganisms may be different [12,25]. Mainly responsible for these properties are gallic, ferulic, and chlorogenic acids but also flavonols such as catechin and epicatechin, found in the extracts tested. The mechanism of their action is to damage the cell membrane of bacteria or fungi, which may disrupt the integrity of the microbial cell membrane and, as a result, lead to the leakage of internal cell components and, ultimately, to its inactivation [11,12,41]. This leads to the inhibition of the growth and multiplication of microorganisms. In addition, the tested compounds in hops, belonging to polyphenols, may inhibit the activity of some enzymes in microorganisms that are necessary for their proper functioning. By blocking these enzymes, these polyphenols can disrupt the metabolic processes of microorganisms, which leads to their weakening or inactivation. Moreover, the mechanism of antimicrobial action is to disrupt the reproductive processes of microorganisms, such as cell division or the formation of new virions. By disrupting these processes, these compounds limit the ability of microorganisms to multiply. Moreover, polyphenols influence gene expression in microorganisms, which leads to changes in their functioning [13,25,41].

Although plant antioxidants are widely used in the food industry, there is a need for further research on their use in pâtés. Areas that require further exploration include, for example, the assessment of the stability of antioxidants in conditions such as the freezing of such pâtés or the impact of variable storage conditions. In addition, in-depth research on the bioavailability and bioactivity of plant antioxidants in the body after eating pâtés is necessary. This research will help understand how antioxidants affect human health after consumption and whether their presence in pâtés translates to health benefits. An important direction of research will be the optimization of the dose and composition of antioxidants from other hop varieties. Research into the optimal dosage and composition of hop antioxidants in pâtés is important to establish the best production practices and ensure the maximum health benefits for consumers. 

## 3. Material and Methods

### 3.1. Materials

#### 3.1.1. Hops

The research material was cones of common hops (*Humulus lupulus* L.), cultivars Magnum and Lubelski, from the plantation in Malice (Kujawsko–Pomorskie province, Kcynia: 52 59052.600 N 17 31020.600 E). The experimental materials were harvested in August 2023. The collected cones were dried using the freeze-drying method (Christ 1-4LSC, Martin Christ Gefriertrocknungsanlagen GmbH—Germany). The basic chemical composition for the cultivars Magnum and Lubelski was, respectively, as follows: 33.43 g/100 g, 35.03 g/100 g, lipids; 4.11 g/100 g 4.58 g/100 g protein; 22.52 g/100 g, 23.33 g/100 g, dietary fiber; and 18.79 g/100 g, 16.61 g/100 g other carbohydrates. The cones were grinded in a Grindomix GM 200 (Retsch, Haan, Germany) for 180 s at 1792× *g* and 21 °C and, afterwards, extracted. 

#### 3.1.2. Extraction Process

The raw material was subjected to water extraction using a three-stages extraction method. The selection of the extraction parameters was based on previous research. The procedure was conducted as follows: 1000 mL of water at 70 °C was combined with 40 g of raw material in three separate portions (400 mL, 300 mL, and 300 mL, respectively) and subjected to extraction three times, each lasting 15 min. Subsequently, the extracts underwent filtration and centrifugation at 67 Gs for 15 min. The resulting fractions were then decanted and filtered using Whatman 1:11 μm filters, and the obtained supernatants were pooled and subjected to freeze-drying. 

#### 3.1.3. Production Process of Liver Pâté

Liver pâtés were produced on a laboratory scale in the Department of Meat Technology, Poznan University of Life Sciences. The raw meat materials came from a local butcher company (from the Greater Poland Voivodeship, Poland). The recipe of the product was as follows: pork class II 43%, fine fat 42%, liver 15%, and broth in the amount of 30% in relation to the meat and fat raw materials. The process included the addition of the following ingredients (per 1 kg of stuffing): salt 1.5%, pepper 0.15%, marjoram 0.05%, and onion 0.4%. The dry spices came from a local chain of retail stores in the city of Poznań. This raw material composition constituted a control sample and a basis for the production of experimental samples. In the experimental trials, 20% of animal fat was replaced with rapeseed oil (OR), and hop extracts were added (Magnum—EChM, and Lubelski—EChL), in amounts of 0.1 and 0.2% (Table 5). Rapeseed oil as a substitute for animal fat in meat products offers numerous advantages. Firstly, due to its neutral color and taste, rapeseed oil does not negatively affect the taste and texture of meat products, while also reducing the saturated fat and calories content.

After thermal treatment at a temperature of 90 °C in a brewing kettle, to a semi-soft state, the raw meat and fat were processed with the addition of rapeseed oil and the spices and hop extracts specified in the recipe (Table 5). At the end of the process, pre-cut liver was dosed to the sample. The final temperature of the stuffing process was 40 °C. 

Then, semi-permeable collagen casings with a diameter of 40 mm were filled with the stuffing. After stuffing, the pâté-type offal sausages were steamed until a temperature of 72 °C was reached at the geometric center of the bar. The temperature inside the pâté was measured with a thermometer on a cable that had been placed inside the sample before it was placed in the smoking and cooking chamber. The sensor was connected to a temperature monitor outside the chamber. After this, the samples were cooled to 4 °C. 

The finished experimental products were placed in a cold room at a temperature of approximately 4 °C. The samples for this research were taken on days 1, 5, 8, 12, and 15 after production.

### 3.2. Methods

#### 3.2.1. Analysis of the Properties of Hop Extracts

The density of the experimental extracts was determined by weighing 1 mL of extract in a weighing vessel and expressed in g/mL. The extraction yield was expressed as %d.w. of the extract sample. 

The quantification of the total polyphenol content was conducted using the Folin–Ciocalteu reagent [42]. The method’s principle involved a spectrophotometric measurement (using Metertek SP-830, Taiwan) of the absorbance of a colored complex. This complex was generated by the reaction between the phenolic groups from the extract and the Folin–Ciocalteu reagent, measured at a wavelength of 765 nm. The outcomes are reported as milligrams of gallic acid equivalent per gram of dry weight of the extract. The content of flavonols and phenolic acids in the hop extracts was assessed based on a procedure previously described by Kobus et al. [42]. The phenolic acids and flavonols were assessed using an Agilent UPLC system equipped with a Bin Pump Infinity DAD 1290 detector. The phenolic acids were detected at wavelengths of 260 nm and 310 nm, while the flavonols were detected at 370 nm. The free radical method employing DPPH (1,1-diphenyl-2-pyrrolhydrazyl) was employed to evaluate the antioxidant capabilities of the analyzed extracts. This method relies on the spectrophotometric measurement of the color of the reaction mixture (using Metertek SP-830, Taiwan). Absorbance was recorded at a wavelength of 517 nm following a 30 min incubation period in the absence of light, at room temperature.

The antioxidant activity was also assessed using the ABTS+ radical test. The activity of the samples was quantified as micromoles of Trolox per gram of dry weight of the extract [42]. First, aqueous solutions of ABTS stock (7 mM) were prepared, along with potassium peroxodisulfate (140 mM). These solutions were then combined to achieve a final concentration of 2.45 mM potassium peroxodisulfate. The mixture was subsequently left at room temperature, in the dark, for 15 h. Prior to analysis, the ABTS•+ cation radical solution was diluted with ethanol to achieve an absorbance of 0.70 ± 0.02 at 734 nm. For the measurement procedure, 100 µL of the analyzed extract was mixed with 2.0 mL of the ABTS•+ cation radical mixture, and absorbance readings were taken after 6 min against the corresponding reagent blank. The analytical results, performed in triplicate and obtained from the calibration curve determined for this standard (ranging from 100 to 1000 µM), were expressed in micromoles of Trolox per gram of dry weight of the extract.

#### 3.2.2. Basic Chemical Composition

The basic chemical composition of the analyzed sausages was determined on the basis of ISO standards. This study took into account indexes such as the water content [43], the protein content [44] (Kjeltec-2200 system, Tecator, Sweden), the fat content [45] (Soxtec-HT6 system, Tecator), and the chloride content, using the Volhard method [46].

#### 3.2.3. Measurement of pH, Redox Potential, and Water Activity a_w_

In our study, the measurement of active acidity (pH) was conducted following the ISO method [47] using a Lab 855 pH meter and a combined pH electrode. 

The redox potential of the analyzed samples was measured with a Lab 855 pH meter using a redox electrode. The water activity of the samples (a_w_) was measured using an equipment AquaLab series 4TE instrument (Pullman, USA), according to the instrument’s instruction manual.

#### 3.2.4. Determination of Primary and Secondary Fat Oxidation Products

##### Peroxide Value (PV)

The determination of the peroxide value (PV), as a primary fat oxidation product, was carried out according to the ISO standard [48]. Lipids from the samples were extracted following the Folch method [49], which utilizes a chloroform–methanol solvent system in a ratio of 2:1. The peroxide values (PV) were calculated using the following equation:

$$\mathrm{PV}=\frac{\left(V_{1}-V_{0}\right)\times T}{m}\times 1000(\mathrm{meq}.O_{2}\times {\mathrm{kg}}^{-1}).$$

*V*_1_—volume of the sodium thiosulfate solution used to titrate the blank (mL);

*V*_0_—volume of the sodium thiosulfate solution used to titrate the sample (mL);

*T*—concentration of sodium thiosulfate used;

*m*—mass of sample portion (g);

1000—conversion factor.

The results were expressed as milliequivalent of active oxygen/kg sample (meq. O_2_ × kg^−1^).

##### Malondialdehyde Content by TBARS Method

The TBARS (2-thiobarbituric acid reactive substances) value, representing a secondary oxidation product, was determined using the distillation method, as described by Tarladgis et al. [50], with some modifications, as described by Pikul et al. [51]. 

During this test, the malondialdehyde (MDA) present in the extracted fat reacts with 2-thiobarbituric acid (TBARS), resulting in the formation of a colored complex. A spectrophotometric analysis of this complex was conducted at 532 nm. The TBARS value was calculated using the following equation:

L_TBARS_= A × K

K—conversion factor equal to 5.5;

A—absorbance value of a given sample. 

The results were expressed as mg malondialdehyde/kg sample (mg MDA × kg^−1^).

#### 3.2.5. Microbiological Analysis

The research procedure included the determination of the following:

- the total number of microorganisms in accordance with the guidelines contained in the ISO standard [52];
- the number of enterococci on Slanetz and Bartley agar, according to the standard PN-A-82055-7:1997 [53];
- the number of rods from the *Enterobacteriaceae* family, as per the guidelines outlined in standards [54,55]— was conducted using a medium known as VRGB (containing bile, neutral red, crystal violet, and glucose).
- the quantification of *Pseudomonas* genus bacteria was carried out following the guidelines outlined in the ISO standard [56], using a CFC medium with agar and cetrimide, fucidin, and cephaloridin.

The results were expressed as logarithm of colony forming units per gram (log_10_ cfu/g).

#### 3.2.6. Chemicals

The quality and purity of all the chemical reagents used in the tests was consistent with the analytical requirements of the quantitative and preparative methods used in this work. The chemical reagents had been obtained from Sigma Aldrich/Merc (USA) and POCH (Poland). The microbiological reagents and media were safe and met the standards and quality requirements for microbiological testing. The reagents and media had been purchased from the company BTL, Poland, Łodź.

#### 3.2.7. Statistical Analysis

The statistical analysis of the obtained results was conducted using the STATISTICA 13.3 and Excel 2010 software. The results presented in this study represent the arithmetic mean derived from two experimental series and three replicates each.

To compare the mean values of the investigated parameters, an analysis of variance for factorial systems was employed, with intergroup differences assessed using Tukey’s test. Statistical significance was determined at a level of α = 0.05.

Furthermore, the relationship between the variables was explored through a linear regression analysis using the equation y = Ax + B, where y represents the dependent variable (the parameter being studied) and x represents the independent variable (such as sample type or storage time). The coefficient A represents the slope of the curve, and B denotes the intercept.

The statistical analysis of changes in the regression slope angle coefficient (A × 10/24 h^−3^) facilitated the examination of dynamic changes occurring over time. 

## 4. Conclusions

This research showed that the extraction efficiency was highest for Lubelski variety hop cones, while it was lowest for Magnum variety hop cones. The Magnum variety cones exhibited a higher phenolic compound content (2.74 ± 0.11 mg/g dry matter) compared to the Lubelska cones (2.27 ± 0.05 mg/g of product). Moreover, the DPPH radical scavenging activity was higher in the extract from the Magnum variety cones (4.21 ± 0.09 mg TE/g d.w.) than in the extract from the Lublin cones (3.87 ± 0.05 mg TE/g d.w.). Similarly, a higher antiradical activity against the ABTS radical was found in the extracts from the Lubelski variety cones compared to those from the Magnum variety cones.

During storage, a statistically significant increase in the pH value was observed in the control sample and in the samples with a 20% replacement of animal fat with rapeseed oil and the addition of Magnum hop extract. However, the addition of Lubuski hop extract led to a decrease in the pH value during the 15-day storage period of the experimental meats. The smallest changes in water activity during storage were observed in the samples with a 20% replacement of animal fat with rapeseed oil and the addition of 0.1% Lubuski hop extract.

The sample with a 20% replacement of animal fat with rapeseed oil and the addition of 0.2% Lubuski hop extract exhibited the lowest peroxide value and TBARS index throughout the storage period. 

The addition of hop extract slowed the growth of total microbial counts in the tested sausages. Furthermore, the content of aerobic microorganisms was lower in the samples with hop extracts compared to the control sample and in the sample with a 20% replacement of animal fat with rapeseed oil. Overall, the addition of hop extract slowed the growth of total microbial counts in the tested sausages.

While plant antioxidants show potential as additives in pâtés, further research is needed to fully understand their impact on product quality and health and develop optimal strategies for their use.

## Figures and Tables

**Table 1:** Chemical characteristics of hop (*Humulus lupulus*) cone extracts: total polyphenol content and antioxidant potential.

Index	Magnum Hop Extract	Lubelski Hop Extract
Density [mg/mL]	1.53 ^b^	1.34 ^a^
Extract yield [%]	12.23 ^a^	14.76 ^b^
Total polyphenols [mg/g d.w. of extract]	2.74 ^a^ ± 0.11	2.27 ^a^ ± 0.05
Chlorogenic acid [μg/g d.w. of extract]	768.32 ^b^ ± 9.8	191.41 ^a^ ± 1.36
Ferulic acid [μg/g d.w. of extract]	0.85 ^a^ ± 0.01	0.68 ^a^ ± 0.02
Vanillic acid [μg/g d.w. of extract]	6.94 ^b^ ± 0.02	2.57 ^a^ ± 0.04
Gallic acid [μg/g d.w. of extract]	14.76 ^a^ ± 0.2	14.33 ^a^ ± 0.11
o-coumaric acid [μg/g d.w. of extract]	29.88 ^a^ ± 0.12	27.43 ^a^ ± 0.47
p-coumaric acid [μg/g d.w. of extract]	51.24 ^a^ ± 0.12	48.51 ^a^ ± 0.31
Cinnamic acid [μg/g d.w. of extract]	6.24 ^a^ ± 0.03	43.87 ^b^ ± 0.29
Syringic acid [μg/g d.w. of extract]	21.44 ^a^ ± 0.14	45.86 ^b^ ± 0.10
p-hydroxybenzoic acid [μg/g d.w. of extract]	71.61 ^a^ ± 0.43	85.66 ^b^ ± 0.31
Caffeic acid [μg/g d.w. of extract]	0.85 ^a^ ± 0.0	2.22 ^b^ ± 0.08
Catechin [μg/g d.w. of extract]	45.43 ^b^ ± 0.21	21.44 ^a^ ± 0.05
Epicatechin [μg/g d.w. of extract]	654.71 ^b^ ± 2.43	287.54 ^a^ ± 0.43
Quercetin [μg/g d.w. of extract]	677.14 ^b^ ± 6.87	378.23 ^a^ ± 4.54
Rutin [μg/g d.w. of extract]	387.81 ^a^ ± 0.36	967.87 ^b^ ± 6.98
Kaempferitrin [μg/g d.w. of extract]	49.9 ^a^ ± 0.0	42.87 ^a^ ± 0.45
DPPH µM Trolox/g d.w.	4.21 ^b^ ± 0.09	3.87 ^a^ ± 0.05
ABTS µM Trolox/g d.w.	1.16 ^a^ ± 0.17	1.25 ^b^ ± 0.05

The mean values in the line marked with different small letters indicate the significance of differences (*p* ≤ 0.05).

**Table 2:** Effect of storage time on changes in physical characteristics in experimental sausages (n = 6), $\overset{-}{x}$ ± sd.

Storage Time (Days)	Sample					
	Control	20% OR	20% OR + 0.1% EChM	20% OR + 0.2% EChM	20% OR + 0.1% EChL	20%OR + 0.2% EChL
**pH LSD A = 0.01; LSD B = 0.01; LSD A × B = 0.02**						
1	6.47 ^cAB^ ± 0.01	6.41 ^aA^ ± 0.01	6.41 ^aAB^ ± 0.00	6.41 ^aA^ ± 0.01	6.44 ^bC^ ± 0.03	6.43 ^bC^ ± 0.01
5	6.48 ^cBC^ ± 0.02	6.42 ^bAB^ ± 0.01	6.40 ^aA^ ± 0.01	6.42 ^bBC^ ± 0.01	6.41 ^abB^ ± 0.02	6.42 ^bBC^ ± 0.01
8	6.46 ^dA^ ± 0.00	6.41 ^bA^ ± 0.01	6.41 ^bAB^ ± 0.03	6.43 ^cC^ ± 0.03	6.39 ^aA^ ± 0.00	6.40 ^abA^ ± 0.01
12	6.49 ^dCD^ ± 0.02	6.43 ^cB^ ± 0.01	6.42 ^bcBC^ ± 0.01	6.42 ^bcBC^ ± 0.01	6.40 ^aAB^ ± 0.01	6.41 ^abAB^ ± 0.01
15	6.50 ^fD^ ± 0.01	6.47 ^eC^ ± 0.04	6.43 ^cC^ ± 0.01	6.45 ^dD^ ± 0.01	6.39 ^aA^ ± 0.01	6.41 b^AB^ ± 0.01
coeff. A × 10/24 h^−3^	2.24	3.65	1.54	2.47	−3.07	−1.38
R^2^	0.64	0.63	0.65	0.77	0.65	0.49
**Redox potential—Eh (mV) LSDA = 15.45; LSD B= 14.11; LSD A × B = 34.55**						
1	171.45 ^dA^ ± 7.19	147.08 ^cA^ ± 7.51	133.00 ^cA^ ± 7.75	115.88 ^bA^ ± 9.77	108.40 ^abA^ ± 6.99	95.90 ^aA^ ± 6.51
5	196.73 ^dB^ ± 6.54	180.73 ^cB^ ± 7.69	161.23 ^bB^ ± 6.16	156.73 ^abB^ ± 8.78	154.63 ^aB^ ± 3.91	143.88 ^aB^ ± 2.04
8	215.50 ^cCD^ ± 8.61	198.08 ^bC^ ± 9.39	179.18 ^aC^ ± 4.63	177.63 ^aC^ ± 3.30	172.70 ^aC^ ± 3.34	169.85 ^aC^ ± 1.87
12	208.95 ^bBC^ ± 3.93	203.75 ^bC^ ± 3.73	196.43 ^abD^ ± 4.75	188.48 ^abCD^ ± 5.52	186.30 ^aCD^ ± 1.86	185.00 ^aD^ ± 2.86
15	224.63 ^cD^ ± 8.67	207.80 ^bC^ ± 7.60	197.33 ^abD^ ± 1.28	200.08 ^abD^ ± 4.57	200.13 ^abD^ ± 2.75	187.58 ^aD^ ± 4.08
coeff. A × 10/24 h^−3^	3375	4156	4714	5744	6181	6463
R^2^	0.83	0.86	0.94	0.92	0.93	0.89
**Water activity LSD A = 0.00; LSD B= 0.00; LSD A × B = 0.01**						
1	0.964 ^dE^ ± 0.01	0.962 ^bE^ ± 0.00	0.956 ^eE^ ± 0.02	0.96 ^aE^ ± 0.00	0.963 ^cE^ ± 0.01	0.961 aE ± 0.00
5	0.954 ^cD^ ± 0.00	0.956 ^dD^ ± 0.00	0.954 ^cD^ ± 0.00	0.953 ^bD^ ± 0.01	0.957 ^eD^ ± 0.00	0.952 ^aD^ ± 0.00
8	0.950 ^dC^ ± 0.00	0.949 ^cC^ ± 0.00	0.948 ^bC^ ± 0.01	0.946 ^aC^ ± 0.00	0.950 ^dC^ ± 0.01	0.948 ^bC^ ± 0.00
12	0.943 ^cB^ ± 0.00	0.943 ^cA^ ± 0.01	0.942 ^bB^ ± 0.00	0.939 ^aA^ ± 0.00	0.946 ^eB^ ± 0.00	0.944 ^dB^ ± 0.00
15	0.941 ^cA^ ± 0.00	0.945 ^dB^ ± 0.00	0.936 ^aA^ ± 0.00	0.938 ^bB^ ± 0.00	0.938 ^bA^ ± 0.00	0.938 ^bA^ ± 0.00
coeff. A × 10/24 h^−3^	−1.67	−1.38	−1.47	−1.77	−1.74	−1.52
R^2^	0.96	0.9	0.95	0.97	0.98	0.98

$\overset{-}{x}$ represents the mean value; n indicates the number of replications; sd denotes the standard deviation; LSD A signifies the least significant difference for the type of sample; and LSD B refers to the least significant difference for storage time. The values represented as a, b, and so forth, followed by their respective standard deviations, indicate statistically significant differences between sample types (*p* ≤ 0.05). Similarly, the values represented as A, B, and so forth, accompanied by their respective standard deviations, denote statistically significant differences in storage time (*p* ≤ 0.05). The linear regression equation is expressed as y = Ax + B, where y represents the dependent variable, x represents the independent variable, A signifies the coefficient for the independent variable per line slope, and B represents the intercept. The coefficient A/24 h indicates changes in the A coefficient during a 24 h storage period. R^2^ denotes the coefficient of determination, with statistical significance set at *p* < 0.05.

**Table 3:** Effect of time on changes in fats of experimental meats (n = 6), $\overset{-}{x}$ ± sd.

Storage Time (Days)	Sample					
	Control	20% OR	20% OR + 0.1% EChM	20% OR + 0.2% EChM	20% OR + 0.1% EChL	20%OR + 0.2% EChL
**Peroxide number (mEq O_2_/kg sample) LSD A = 0.02; LSD B = 0.02; LSD A × B = 0.05**						
1	0.21 ^cA^ ± 0.02	0.21 ^cA^ ± 0.02	0.18 ^abA^ ± 0.03	0.18 ^abA^ ± 0.03	0.16 ^aA^ ± 0.02	0.18 ^abA^ ± 0.03
5	0.33 ^dB^ ± 0.06	0.23 ^bcA^ ± 0.03	0.24 ^cB^ ± 0.02	0.23 ^bcB^ ± 0.03	0.20 ^aB^ ± 0.04	0.21 ^abB^ ± 0.03
8	0.35 ^cB^ ± 0.09	0.36 ^cB^ ± 0.05	0.29 ^bC^ ± 0.05	0.26 ^aC^ ± 0.02	0.28 ^abC^ ± 0.03	0.26 ^aC^ ± 0.05
12	0.43 ^cC^ ± 0.03	0.41 ^cC^ ± 0.05	0.34 ^bD^ ± 0.02	0.31 ^aD^ ± 0.02	0.30 ^aC^ ± 0.04	0.29 ^aD^ ± 0.02
15	0.53 ^eD^ ± 0.06	0.46 ^dD^ ± 0.03	0.39 ^cE^ ± 0.03	0.36 ^bE^ ± 0.02	0.36 ^bD^ ± 0.02	0.33 ^aE^ ± 0.05
coeff. A × 10/24 h^−3^	21	19	15	13	14	11
R^2^	0.97	0.94	0.99	0.99	0.96	0.99
**TBARS (mg/kg of sample) LSD A = 0.04; LSD B = 0.04; LSD A × B = 0.10**						
1	1.07 ^bcA^ ± 0.08	1.10 ^cA^ ± 0.06	1.04 ^abA^ ± 0.05	1.07 ^bcA^ ± 0.05	1.05 ^abA^ ± 0.08	1.02 ^aA^ ± 0.08
5	1.28 ^cB^ ± 0.14	1.24 ^cB^ ± 0.12	1.06 ^aA^ ± 0.07	1.10 ^aA^ ± 0.07	1.15 ^bB^ ± 0.08	1.09 ^aB^ ± 0.07
8	1.41 ^dC^ ± 0.11	1.56 ^eC^ ± 0.11	1.36 ^cB^ ± 0.05	1.18 ^aB^ ± 0.05	1.22 ^abC^ ± 0.03	1.23 ^bC^ ± 0.04
12	1.53 ^cD^ ± 0.06	1.66 ^dD^ ± 0.05	1.49 ^bcC^ ± 0.04	1.26 ^aC^ ± 0.04	1.46 ^bD^ ± 0.03	1.27 ^aC^ ± 0.04
15	1.85 ^dE^ ± 0.06	1.87 ^dE^ ± 0.04	1.63 ^cD^ ± 0.10	1.50 ^bD^ ± 0.07	1.65 ^cE^ ± 0.04	1.43 ^aD^ ± 0.04
coeff. A × 10/24 h^−3^	51	55	45	28	43	28
R^2^	0.96	0.97	0.94	0.86	0.95	0.95

$\overset{-}{x}$ represents the mean value; n indicates the number of replications; sd denotes the standard deviation; LSD A signifies the least significant difference for the type of sample; and LSD B refers to the least significant difference for storage time. The values represented as a, b, and so forth, followed by their respective standard deviations, indicate statistically significant differences between sample types (*p* ≤ 0.05). Similarly, the values represented as A, B, and so forth, accompanied by their respective standard deviations, denote statistically significant differences in storage time (*p* ≤ 0.05). The linear regression equation is expressed as y = Ax + B, where y represents the dependent variable, x represents the independent variable, A signifies the coefficient for the independent variable per line slope, and B represents the intercept. The coefficient A/24 h indicates changes in the A coefficient during a 24 h storage period. R^2^ denotes the coefficient of determination, with statistical significance set at *p* < 0.05.

**Table 4:** Effect of storage time on changes in microbial quality in experimental sausages (n = 6), $\overset{-}{x}$ ± sd.

Storage Time (Days)	Sample					
	Control	20% OR	20% OR + 0.1% EChM	20% OR + 0.2% EChM	20% OR + 0.1% EChL	20%OR + 0.2% EChL
**Total count of mesophilic bacteria (log_10_ cfu/g) LSD A = 0.18; LSD B = 0.16; LSD A × B = 0.40**						
1	1.74 ^dA^ ± 0.16	1.59 ^cA^ ± 0.25	1.24 ^aA^ ± 0.26	1.38 ^bA^ ± 0.05	1.29 ^abA^ ± 0.10	1.36 ^bA^ ± 0.04
5	1.79 ^cA^ ± 0.08	1.48 ^abA^ ± 0.15	1.40 ^aA^ ± 0.35	1.44 ^abA^ ± 0.29	1.50 ^abB^ ± 0.26	1.40 ^aA^ ± 0.14
8	2.37 ^cB^ ± 0.39	2.36 ^cB^ ± 0.13	2.31 ^bB^ ± 0.32	2.40 ^cB^ ± 0.11	2.17 ^abC^ ± 0.12	2.10 ^aB^ ± 0.22
12	3.63 ^cC^ ± 0.35	3.85 ^dC^ ± 0.27	2.86 ^bC^ ± 0.10	2.62 ^aC^ ± 0.12	2.71 ^abC^ ± 0.13	2.57 ^aC^ ± 0.36
15	4.88 ^cD^ ± 0.30	5.04 ^dD^ ± 0.06	3.43 ^bD^ ± 0.32	3.06 ^aD^ ± 0.25	3.04 ^aD^ ± 0.26	2.96 ^aD^ ± 0.15
coeff. A × 10/24 h^−3^	230	263	166	128	134	124
R^2^	0.89	0.89	0.96	0.92	0.97	0.94
***Pseudomonas* (log_10_ cfu/g) LSD A = 0.20; LSD B= 0.19; LSD A × B = 0.46**						
1	2.46 ^dA^ ± 0.25	1.73 ^aA^ ± 0.29	2.05 ^cA^ ± 0.30	1.91 ^abA^ ± 0.14	1.98 ^bcA^ ± 0.23	1.85 ^abcA^ ± 0.18
5	3.19 ^aB^ ± 0.24	3.35 ^aC^ ± 0.26	3.27 ^aB^ ± 0.20	3.31 ^aB^ ± 0.23	3.36 ^aB^ ± 0.24	3.25 ^aB^ ± 0.13
8	2.60 ^aA^ ± 0.24	2.42 ^aB^ ± 0.35	3.54 ^bC^ ± 0.20	3.75 ^cD^ ± 0.10	3.72 ^bcC^ ± 0.04	3.61 ^bcC^ ± 0.28
12	3.85 ^cD^ ± 0.12	3.52 ^abCD^ ± 0.17	3.86 ^cD^ ± 0.07	3.56 ^bCD^ ± 0.22	3.83 ^cC^ ± 0.06	3.33 ^aB^ ± 0.17
15	3.67 ^cC^ ± 0.09	3.67 ^cD^ ± 0.09	3.64 ^bcC^ ± 0.13	3.52 ^abC^ ± 0.17	3.73 ^cC^ ± 0.10	3.42 ^aBC^ ± 0.15
coeff. A × 10/24 h^−3^	90	120	110	101	115	94
R^2^	0.66	0.63	0.72	0.56	0.69	0.53

$\overset{-}{x}$ represents the mean value; n indicates the number of replications; sd denotes the standard deviation; LSD A signifies the least significant difference for the type of sample; and LSD B refers to the least significant difference for storage time. The values represented as a, b, and so forth, followed by their respective standard deviations, indicate statistically significant differences between sample types (*p* ≤ 0.05). Similarly, the values represented as A, B, and so forth, accompanied by their respective standard deviations, denote statistically significant differences in storage time (*p* ≤ 0.05). The linear regression equation is expressed as y = Ax + B, where y represents the dependent variable, x represents the independent variable, A signifies the coefficient for the independent variable per line slope, and B represents the intercept. The coefficient A/24 h indicates changes in the A coefficient during a 24 h storage period. R^2^ denotes the coefficient of determination, with statistical significance set at *p* < 0.05.

**Table 5:** Raw material composition of the experimental sausages [g kg^−1^].

Trials	Class II Pork	Fine Fat	Rapeseed Oil	Pork Liver	Spices	Hops Extract	
						Magnum	Lubelski
	g kg^−1^						
Control	430	420	-	150	21		-
20% OR	430	336	84	150	21		-
20% OR + 0.1% EChM	430	336	84	150	21	1	-
20% OR + 0.2% EChM	430	336	84	150	21	2	-
20% OR+ 0.1% EChL	430	336	84	150	21	-	1
20%OR + 0.2% EChL	430	336	84	150	21	-	2

## Data Availability

The data can be shared up on request.
